# A case report of labyrinthine infarction: a ‘central’ cause of vertigo with ‘peripheral’ presentation

**DOI:** 10.1097/MS9.0000000000002579

**Published:** 2024-09-24

**Authors:** Khadija El Bouhmadi, Safa Darouich, Myriam Youbi, Said Anajar, Mustapha Essaadi, Khalid Snoussi, Amal Hajjij

**Affiliations:** Mohammed VI University of Sciences and Health (UM6SS) Vertigo Center, Department of Otolaryngology and Head and Neck Surgery Cheikh Khalifa International University Hospital, Casablanca, Morocco

**Keywords:** case report, labyrinthine infarction, MRI, nystagmus, vertigo

## Abstract

**Introduction and Importance:**

The inner ear is considered an ‘end organ’ since its vascular supply comes from one terminal artery, making the labyrinth especially vulnerable to ischemia, causing loss of auditory and vestibular function with variable clinical patterns according to the different arterial distribution in the inner ear and which vascular branches are concerned by the embolism.

**Case Presentation:**

We report a misleading case of central vascular vertigo caused by a labyrinthine infarction resulting from an embolic vertebral artery, which manifested in a typical peripheral clinical presentation mimicking a vestibular neuritis.

**Clinical Discussion:**

Vertigo is the result of asymmetrical responses from the vestibules of both ears resulting from any disruption along the complex vestibular pathways, whether peripheral or central. The recognition of the origin of an acute isolated vertigo is fundamental since the therapeutic strategy and prognosis differ, but it can be challenging in the absence of neurological signs, especially when the clinical pattern involves only the vestibular part of the labyrinth.

**Conclusion:**

The diagnosis strategy should consider the patient vascular risk factors and the clinical bedside tests with diffusion-weighted magnetic resonance imaging (MRI). Then, the management of these patients requires pluridisciplinary cooperation with early vestibular rehabilitation.

## Introduction

Highlights

- The labyrinth is especially vulnerable to ischemia, causing loss of auditory and vestibular function with variable clinical patterns according to which vascular branches are concerned by the embolism.
- The recognition of the origin of an acute isolated vertigo is fundamental since the therapeutic strategy and prognosis differ.
- The differentiation between the central and peripheral origin of vertigo can be challenging in the absence of neurological and auditory signs.
- The diagnosis strategy should consider the patient vascular risk factors and the bedside tests associated with diffusion-weighted MRI.
- The management of these patients requires pluridisciplinary cooperation with early vestibular rehabilitation.

The inner ear is considered an ‘end organ’ since its vascular supply comes from one terminal artery with minimal collaterals from the otic capsule named ‘the labyrinthine artery’^1,2^. Thus, the labyrinth is especially vulnerable to ischemia, and even transient ischemia can cause permanent damage resulting in loss of auditory and vestibular function with variable clinical patterns according to the different arterial distribution in the inner ear and which vascular branches are concerned by the embolism^2,3^.

Vertigo is the result of asymmetrical responses from the vestibules of both ears resulting from any disruption along the complex vestibular pathways, whether peripheral or central^4^. The recognition of the origin of an acute isolated vertigo is fundamental since the treatment and prognosis differ. Then, the diagnosis strategy should consider the patient vascular risk factors and the bedside tests associated with diffusion-weighted MRI^2^.

We report a misleading case of central vascular vertigo caused by a labyrinthine infarction mimicking peripheral vestibular neuritis, showing how challenging the diagnosis can be in the absence of neurological signs, especially when the clinical pattern involves only the vestibular part of the labyrinth.

## Presentation of the case

We report the case of a 59-year-old man who presented for 48 h acute spontaneous severe rotational vertigo, significantly increased by head movements with intense dizziness, nausea, and vomiting. No current hearing loss, tinnitus, or neurological signs such as headaches, facial palsy, or paresthesia of the upper and lower extremities were reported. Nevertheless, the patient had a history of acute vertigo back in 2013 with left sudden sensorineural hearing loss not explored nor rehabilitated and was under valsartan and hydrochlorothiazide combination for arterial hypertension.

We received in the Emergency Room (ER) a patient in a wheelchair, eyes closed, but eventually able to stand alone with a left deviation at the Romberg test and a tendency to fall. The bedside examination found one direction right horizontal-torsional spontaneous nystagmus bare eyes, increasing under videonystagmoscopy (VNS) and in the Head Shaking Test (HST) and decreasing with fixation, associated with saccadic pursuit. The Rapid Head Impulse Test showed ‘catch-up’ saccades in the left head impulses. No skew deviation was found and the head CT scan returned normal.

The diagnosis of left vestibular neuritis was retained, and the treatment was started. The patient received an intravenous route for high doses of corticosteroids (120 mg of methylprednisolone per day) and antiemetic therapy (10 mg of metoclopramide 3 times a day) associated with 500 mg of acetylleucine 3 times a day.

The evolution was marked by rapid improvement of his vestibular symptoms, with decreasing vertigo and spontaneous nystagmus. However, 24 h later, the patient complained of isolated moderate headaches with no aura, blurry vision, or other cranial nerve lesions.

**Figure 1:**
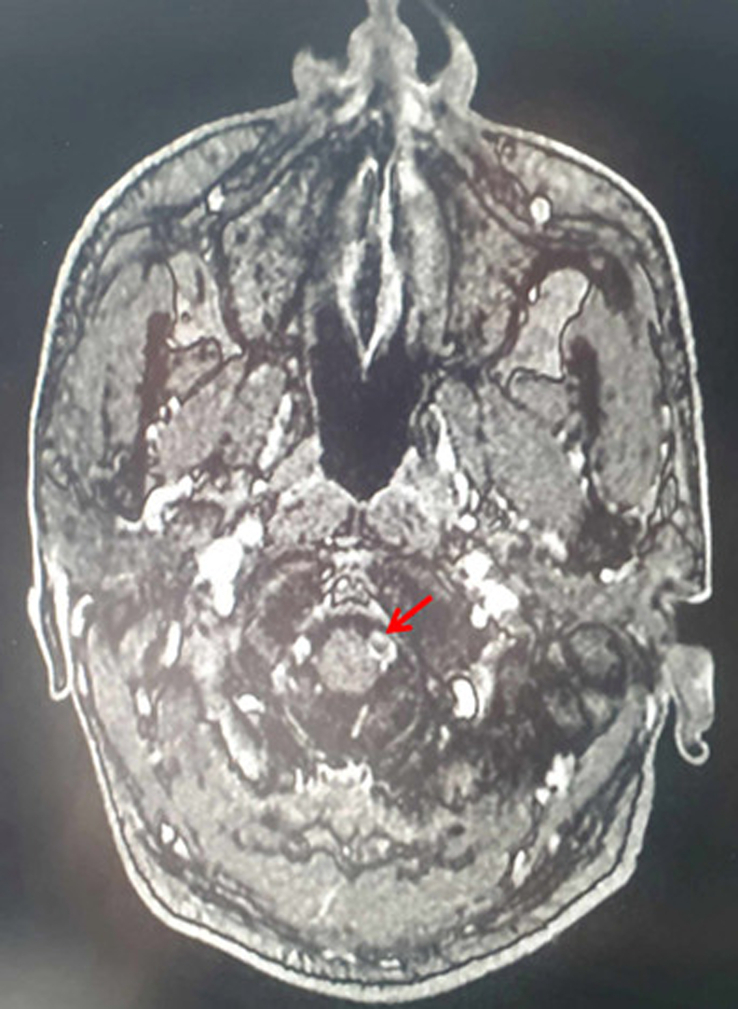
Obliterated left vertebral artery (red arrow) in MRI angiography sequence.

Cerebral MRI was immediately indicated and showed multiple acute ischemic strokes in the territories of the posterior cerebral arteries and the left vertebral artery, with no opacification at the angiographic sequences (Fig. 1). The embolic vertebral artery caused labyrinthine infarction responsible for the acute left vestibular deficit.

**Figure 2:**
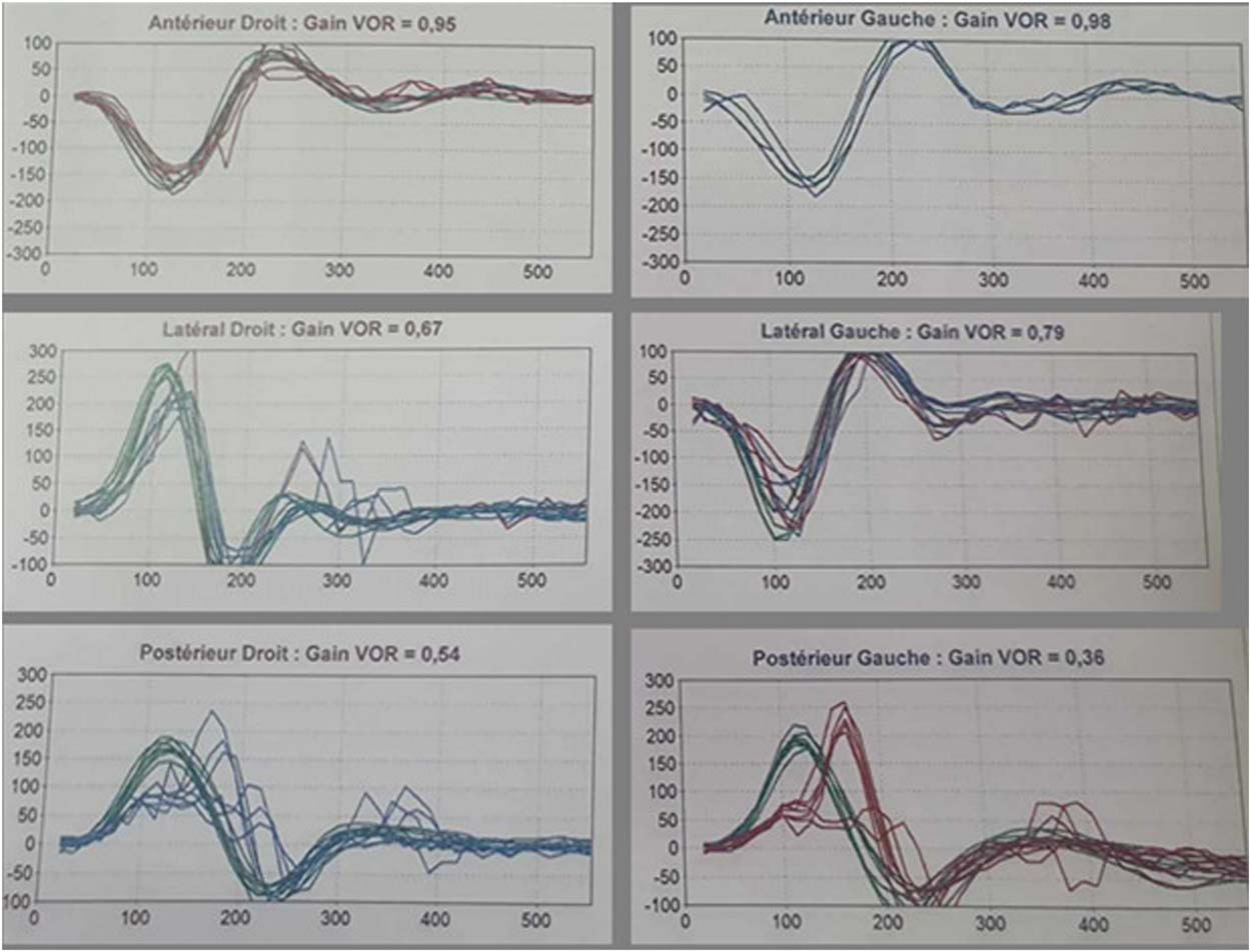
Low VOR gains illustrated by catch-up saccades for the lateral and posterior SCC.

The patient was then transferred to the neurological intensive care unit and treated with anticoagulant. We received the patient 10 days later walking, helped by a rollator walker, with no vertigo. He kept the left deviation in the Romberg test, and the right horizontal-torsional nystagmus was less intense, only appearing under VNS. Vestibular investigations included a Video Head Impulse Test (VHIT) to assess high frequencies, which showed variable low VOR gains in the horizontal semicircular canals (SCC) (0.67 in the right and 0.79 in the left) and in the posterior SCC (0.54 in the right and 0.36 in the left) with normal gains for the anterior SCC (Fig. 2).

**Figure 3:**
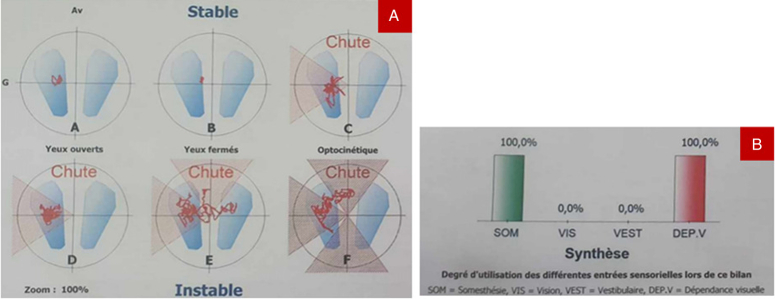
(A) Posturography results with falls since condition C. (B) Sensory analysis with absent visual and vestibular inputs and 100% visual dependency.

In posturography (Multitest platform Framiral), the center of foot pressure was moved to the left with falls since condition C with sway-referenced visual surround and high postural instability index. The vestibular input in the sensory analysis was completely absent (0%) with visual dependency at 100% (Fig. 3).

**Figure 4:**
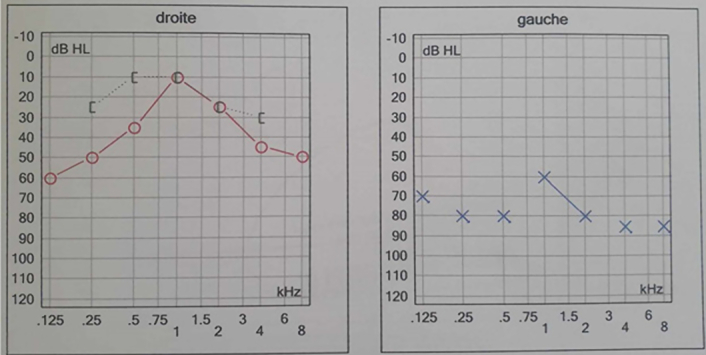
Subtotal left deafness on tonal audiometry.

Auditory evaluation by tonal audiometry found severe left sensorineural hearing loss (Fig. 4). Based on these results, vestibular rehabilitation therapy was started with major clinical improvement after five sessions and the patient returned to his teaching job in 6 weeks.

Our work has been reported in line with the SCARE 2023 criteria^5^.

## Discussion

The first systematic descriptions of vascular irrigation of the human cochlea go back to 1887 by Schwalbe^6^, followed by Eichler in 1892^7^ and Siebenmann in 1894^8^. In 1923, using intravascular dye injections, Nabeya found that the vascular supply of the inner ear comes from one terminal artery that he named ‘the labyrinthine artery’^1^.

**Figure 5:**
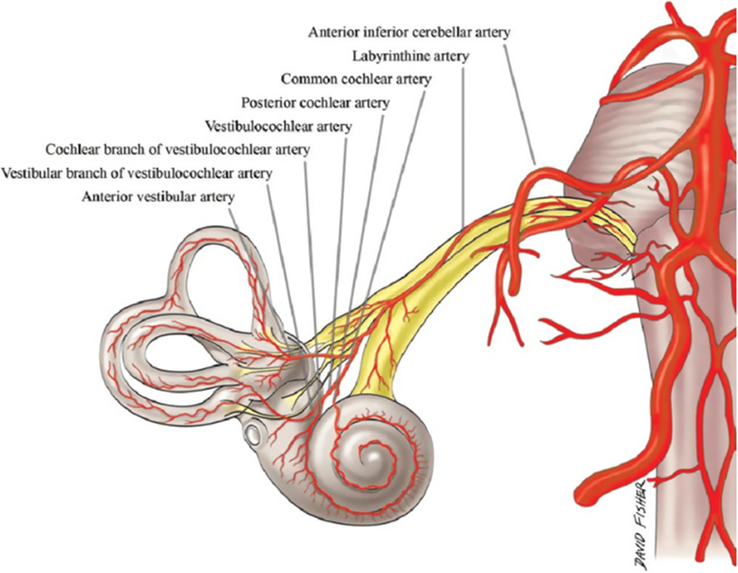
Arterial supply of the inner ear^12^.

Indeed, the labyrinth irrigation comes from the lower part of the Willis circle since it is supplied by the labyrinthine artery (LA), also called the internal auditory artery (IAA), which branches most commonly (83.6%) from the anterior inferior cerebellar artery (AICA) or occasionally (12.3%) from the basilar artery, both arising from the vertebral artery (VA), itself a branch of the subclavian artery. Then, the IAA follows the facial and the vestibulocochlear nerves through the internal auditory canal, where it divides into three branches, tracing a path across the perforated bony wall of the fundus and reaching the membranous labyrinth^2,9^. The anterior vestibular artery (AVA) supplies most of the utricle, the vestibular nerve, and part of the SCC. The cochlear artery (CA) is the main cochlear vasculature. The vestibulocochlear artery (VCA) is separated into two branches running in opposite directions; the vestibular branch supplies the vestibule and the SCC, while the cochlear branch follows a spiral pathway along the scala vestibule and anastomoses with the CA^2,9,10^. The vascularization of the vestibular central pathways depends on the AICA, supplying the flocculus of the cerebellum; and the posterior inferior cerebellar artery (PICA) arising from the vertebral artery and supplying the nodulus, the inferior cerebellar peduncle, the vestibular nuclei, and the spinal tract and nucleus of the fifth cranial nerve^11^. (Fig. 5).

Since the IAA is a terminal artery with minimal collaterals, the labyrinth is especially vulnerable to ischemia and transient ischemia can cause permanent damage. Effectively, cochlear electrical activity deteriorates within 60 s of blood flow interruption and may not return to normal if the obstruction exceeds 30 min; with higher vulnerability of the apical region of the cochlea explaining the common low-frequency hearing loss^2,3^. However, the vestibular end organs are relatively resistant. Also, each compartment of the labyrinth may be selectively involved, resulting in multiple clinical patterns according to the different arterial distribution in the inner ear and which IAA branches are concerned by the embolism: the ischemic lesion can concern all cochlear and vestibular end organs, or the cochlea, saccule, and the posterior canal ampulla, or the utricle, part of the saccule, and the anterior and horizontal canal ampullae, without cochlear involvement^3,13^.

About 20% of ischemic events concern the posterior vertebrobasilar territory. IAA infarction mostly occurs due to thrombotic narrowing of the AICA itself or in the basilar artery at the orifice of the AICA; rarely, it results from an embolic vertebral artery, as reported in our case^2^.

Vertigo is the result of asymmetrical responses from the vestibules of both ears resulting from any disruption along the complex vestibular pathways, whether peripheral, which accounts for over 90% of all causes of vertigo or central^4^. Thus, recognition of the origin of an isolated vertigo is fundamental since the therapeutic strategy and prognosis differ. When central causes usually need specific management, with a significant increase in morbidity and mortality if an acute stroke is lately or misdiagnosed, overdiagnosing vascular vertigo leads to unnecessary expensive work-ups. However, the central signs, highly specific but slightly sensitive, do not appear in all patients, so the differentiation can be challenging^2,11^. Indeed, major peripheral differential diagnoses in the emergency setting are caused by presumed viral vestibular neuritis, rarely by labyrinthitis, which is characterized by associated auditory symptoms^4,14^.

Thus, anamnesis has a key role. The vascular origin of acute vertigo should be considered in older patients, particularly when there is a history of stroke or known vascular risk factors such as smoking, diabetes mellitus, hypertension, hypercholesterolemia, and cardiovascular disease^2,15^. These risk factors are variable and more specific to certain stroke subtypes; while hypertension is majorly implicated in both hemorrhagic and ischemic stroke since it contributes to atherosclerotic disease, hyperlipidemia is linked to ischemic lesions, and atrial fibrillation is a risk factor for cardioembolic stroke^16^.

Since the IAA supplies the cochlea and the vestibular, its occlusion causes loss of auditory and vestibular function, which misled us in our case according to the fact that our patient already had profound deafness in that ear^2^.

On a clinical level, the HINTS exam, which is a cluster of three bedside clinical tests (head impulse test, nystagmus, and skew deviation), is considered the best bedside test to separate the central from the peripheral causes of vertigo and should be performed every time a central vertigo is suspected, being valid when the symptomatology is still ongoing and they are considered more sensitive to stroke than early MRI imaging^2,4^. Indeed, in stroke, the nystagmus is pure vertical or rotational, changing direction with the gaze, with no modification at fixation. The patient usually reports difficulty standing which may be associated with neurological signs such as stuttering and paresthesia of the ipsilateral face and contralateral upper and lower extremities^2^.

On the hand, ‘vestibular pseudoneuritis’ (VPN) is a particular clinical entity characterized by lesions of the labyrinthine, the entry zone of the eighth nerve, pontomedullary brainstem, or cerebellum resulting from isolated infarctions or multiple sclerosis plaques. The clinical presentation of VPN may mimic vestibular neuritis, such as in our case, and the differentiation between the two diagnoses is very meticulous and relevant since patients with vascular VPN may suffer a second preventable infarction of the brainstem and/or cerebellum and would need further investigations and management, but very challenging when the patient has no non-vestibular signs^2,14^. Indeed, Cnyrim *et al*.^14^ demonstrated by multivariate logistic regression that the combination of five signs (gaze-evoked nystagmus, saccadic pursuit, head thrust test, skew deviation, subjective visual vertical) increases the sensitivity and specificity of bedside tests to 92% in VPN diagnosis.

Even if brain CT is quicker and easily available, it is known as less accurate in the early diagnosis of acute ischemic lesions within the posterior fossa; thus, MRI with diffusion imaging is considered the golden standard in cases of isolated vertigo secondary to vascular ischemic lesions^2,15^. However, it has been reported in a recent systemic review that the MRI is possibly misleading up to at least 48 h after the beginning of the symptoms^2^. In smaller strokes (<1 cm), early MRI diagnosis can fail up to 50% of the cases^15^.

The vertigo and vomiting can be managed with symptomatic medications, and vestibular rehabilitation should begin early. Also, the underlying cause and risk factors should be treated effectively. Stroke management may need close monitoring in the intensive care unit for the development of malignant ischemic edema, which may be delayed at times for several days. Also, treatment may require fibrinolytic or antiaggregant drugs adjusted to the kidney or hepatic impairment started as soon as possible or different percutaneous transcatheter interventions or surgical procedures (thrombectomy, external ventricular drain, resection of necrotic tissue) with good post outcome^3,15^.

Concerning the hypothesis that the corticosteroid treatment provoked the stroke, it majorly lacks evidence in our case, especially since the patient noticed clinical improvement after corticosteroid injections with a notable decrease in his vertigo and spontaneous nystagmus. Also, the cases we found in the literature concerned higher doses of corticosteroid (IV 1 g/day for 3 days followed by oral 1 mg/kg then gradually reduced of 5 mg per day till a dose of 5 mg per day), described in a patient with a clinical context (chronic inflammatory bowel disease and unknown paroxysmal atrial fibrillation)^17^. In our case, the vascular embolism happened after only one dose of 120 mg of methylprednisolone in a patient with no other vascular factor than arterial hypertension under treatment and a monitored cardiac sinus rhythm throughout his hospitalization.

Usually, the follow-up shows resolution of the vertigo, nystagmus, and autonomic manifestations over days to weeks; however, the hearing loss typically remains and canal paresis normalizes within 5 years^3^. The overall prognosis and the risk of recurrence depend on the underlying etiology. Associated brainstem or cerebellar infarcts worsen the prognosis, while their absence with normal brain imaging decreases considerably the risk of stroke recurrence. The possible late effects may include benign paroxysmal positioning vertigo since utricle ischemia results in the displacement of otoconia from the macula to the still-functioning posterior SCC^3^.

The main limitations of our case study include its retrospective nature and the lack of important laboratory work to rule out the other vascular risk factors.

## Conclusion

The recognition of the origin of isolated vertigo is fundamental since the therapeutic strategy and prognosis differ, but it can be challenging in the absence of neurological signs, especially when the ischemic lesion involves only the vestibular part of the inner ear. The diagnosis strategy should consider the patient vascular risk factors and the HINTS bedside tests with diffusion-weighted MRI. Then, the management of these patients requires pluridisciplinary cooperation with early vestibular rehabilitation.

## Ethical approval

This work has been approved by our department’s ethical committee. All the patients gave their consent for the surgery and for the follow-up that led to this study.

## Consent

Written informed consent was obtained from the patient for publication and any accompanying images. A copy of the written consent form is available for review by the editor-in-chief of this journal upon request.

## Source of funding

None.

## Author contribution

E.B.K.: corresponding author, patient follow-up, and writing the paper; D.S. and Y.M.: patient follow-up; A.S., E.M., S.K., and H.A.: correction of the paper.

## Conflicts of interest disclosure

The authors have no conflicts of interest.

## Research registration unique identifying number (UIN)

Not needed since the article is a descriptive case report.

## Guarantor

El Bouhmadi Khadija.

## Data availability statement

All the data are publicly available.

## Provenance and peer review

Not commissioned, externally peer-reviewed.
